# Abnormal Pap Follow-Up among Criminal-Legal Involved Women in Three U.S. Cities

**DOI:** 10.3390/ijerph18126556

**Published:** 2021-06-18

**Authors:** Chelsea Salyer, Ashlyn Lipnicky, Meredith Bagwell-Gray, Jennifer Lorvick, Karen Cropsey, Megha Ramaswamy

**Affiliations:** 1 Division of Gynecologic Oncology, University of Kansas, Kansas City, KS 66160, USA; csalyer2@kumc.edu; 2 Department of Population Health, University of Kansas, Kansas City, KS 66160, USA; alipnicky@kumc.edu; 3 School of Social Welfare, University of Kansas, Lawrence, KS 66045, USA; meredith.bagwell-gray@ku.edu; 4 RTI International Community Health and Implementation Research Program, Berkeley, CA 94704, USA; jlorvick@rti.org; 5 Department of Psychiatry, University of Alabama, Birmingham, AL 35294, USA; kcropsey@uabmc.edu

**Keywords:** cervical cancer disparities, criminal-legal involved women, abnormal Pap follow-up

## Abstract

Criminal-legal involved women experience significant barriers to preventive cervical care, and consequently there is a higher incidence of cervical cancer in this population. The purpose of this study is to identify variables that may facilitate abnormal Pap follow-up among criminal-legal involved women living in community settings. The study included *n* = 510 women with criminal-legal histories, from three U.S. cities—Birmingham, AL; Kansas City, KS/MO; Oakland, CA. Participants completed a 288-item survey, with questions related to demographics, social advantages, provider communication, and reasons for missing follow-up care. There were *n* = 58 women who reported abnormal Pap testing, and *n* = 40 (69%) received follow-up care. Most women received either repeat Pap/HPV testing (*n* = 15, 38%), or colposcopy and/or biopsy (*n* = 14, 35%). Women who did not follow-up (*n* = 15, 26%) cited that they forgot (*n* = 8, 53%), were uninsured (*n* = 3, 20%), or were reincarcerated (*n* = 3, 20%). In a multivariate analysis, both having a primary care provider (OR 4.6, 95% CI 1.3–16.0) and receiving specific provider communication about follow-up (OR 3.8, 95% CI 1.1–13.2) were independent predictors for abnormal Pap follow-up. Interventions that offer linkages to providers in the community or ensure abnormal Pap care plans are communicated effectively may mitigate the disparate incidence of cervical cancer among criminal-legal involved women.

## 1. Introduction

Cervical cancer is preventable with routine Papanicolaou (Pap) testing and dysplasia treatment, yet women with criminal-legal histories are five times more likely to develop cervical cancer in their lifetimes [1,2]. There are nearly two million U.S. women with a history of criminal-legal involvement, which includes women who are housed in correctional facilities, supervised under probation, parole, or other community-based monitoring systems, or women who are previously incarcerated without ongoing oversight [3]. Surveys of incarcerated women in the U.S. report that 67–84% of criminal-legal involved women have completed Pap testing within three years, which is comparable to the Pap testing rates cited for all women [4,5,6]. However, after receiving an abnormal Pap result, only 21–66% of criminal-legal involved women obtain the recommended follow-up—which is much lower than the 90% follow-up rate observed among women in the general population [7,8,9,10]. While criminal-legal involved women may have access to Pap testing, they have higher rates of cervical dysplasia, and are less likely to receive the necessary surveillance and treatments that can prevent the progression to cervical cancer. 

There have been efforts to expand preventive cervical care within jail and prison settings, but this approach has limitations. In the U.S., follow-up after an abnormal Pap diagnosis from cytology testing typically involves receiving human papillomavirus (HPV) typing and/or colposcopy, with or without a cervical biopsy. In the cases of high-grade dysplasia, providers may advise women to have additional preventive treatments, including ablation procedures, loop electrical excision procedure (LEEP), or hysterectomy. Screening with primary HPV testing is now recommended in the U.S., but this has not yet been widely adopted as standard practice [11]. Most jails and prisons are not equipped to provide even basic gynecologic care, much less offer testing and procedures that require access to specialized diagnostic equipment [12]. Furthermore, most women are released within a short period of time and do not have the opportunity to participate in jail-based cervical health programs [9,13]. 

Following release from jail or prison, criminal-legal involved women encounter significant barriers to cervical care—they must obtain health insurance and find a primary care provider, while also securing income, housing, food, and other basic needs [8,14,15]. Barriers that limit access to preventive cervical care have been well studied, but there are less data on factors that will facilitate abnormal Pap follow-up. In studies of incarcerated women, abnormal Pap follow-up has been associated with lesion severity, duration of incarceration, and demographic characteristics [9,10,16]. In those studies, women who were incarcerated for a longer period after the abnormal Pap result were more likely to comply with the follow-up, as were women with fewer aliases, lower educational attainment, and a diagnosis of low-grade squamous intraepithelial lesion (LSIL), or higher [9,10,16]. For women without criminal-legal histories, both access to a primary care provider and aggressive follow-up with tailored provider communication, case management, and universal colposcopy predicted adherence to abnormal Pap follow-up recommendations [17,18,19]. Given that 80% of criminal-legal involved women live outside of carceral settings, it is important to understand what variables enable women to obtain follow-up care for abnormal Pap results in their communities [20]. The purpose of this study was to identify predictors for abnormal Pap follow-up among community-based criminal-legal involved women from three U.S. cities—Birmingham, AL; Kansas City, KS/MO; and Oakland, CA. 

## 2. Materials and Methods

This is a cross-sectional study of criminal-legal involved women with abnormal Pap testing from three U.S. cities. Data were obtained from a baseline survey administered from March 2019 to June 2020 for the tri-city cervical cancer prevention study, which is an ongoing longitudinal (2019–2024) study with criminal-legal involved women who are living in community settings. All participants were women age 18 or older and recruited from cohorts of women in ongoing, separate studies at each location. The three cohorts were merged to provide the investigative team with access to existing groups of hard-to-reach women and follow a robust, multi-site cohort. All women were living outside of the jail/prison setting; Oakland women were on probation and Kansas City women were all recently released from city/county jails without any ongoing oversight. Birmingham women were receiving ongoing monitoring through an outpatient case management facility, which included supervision under diversion programs such as drug court, mental health court, probation, and life after prison/parole. The women at each study location were under varying degrees of legal-based oversight, but they were all released to live in community settings by their prior correctional facility. 

Participants were given a 288-item survey that investigated preventive cervical care and other health behaviors. The survey included open-ended and multiple-choice questions informed by the National Cancer Institute Health Information National Trends Survey (NCI HINTS), cervical cancer section [21]. Information on Pap testing and follow-up care were designed using the American College of Obstetrics and Gynecologists (ACOG), American Society for Colposcopy and Cervical Pathology (ASCCP), and the U.S. Preventive Service Task Force (USPSTF) guidelines on cervical cancer screening and follow-up [22,23,24]. At the time of survey development, the recommended strategies for cervical cancer screening included the following: (1) cervical cytology alone every three years for women aged 21–29, (2) cervical cytology alone every three years for women aged 30–65, or (3) cervical cytology and HPV co-testing every five years for women aged 30–65 [22,23,24]. In 2018, the USPSTF updated their guidelines to include primary HPV testing every five years as the optimal screening approach for women aged 30–65, which has been recommended by many international cervical cancer screening organizations including the World Health Organization [23,25]. As of 2021, ACOG has also recommended HPV screening alone as the preferred method for cervical cancer screening [11]. However, at the time of survey administration, this approach was not yet widely adopted by U.S. practitioners so the survey did not include HPV testing alone as a possible response for type of cervical cancer screening. For abnormal Pap follow-up care, ACOG, USPSTF, and ASCCP recommend that women with low-grade Pap results have either HPV co-testing, a repeat Pap in one year, or immediate colposcopy depending on their age and HPV result (when available). Women with suspected high-grade lesions are advised to have HPV testing (if not already obtained) and either colposcopy with biopsy, or excision with a loop electrosurgical excision (LEEP) procedure depending on age and HPV status. Women with high-grade lesions may also elect to proceed with a hysterectomy if they do not desire future childbearing [22,23,24]. 

To assess participant’s cervical cancer screening status, women were asked multiple-choice questions about the timing of their last Pap test and whether they had an HPV co-test. Data on Pap timing were then stratified by time intervals of less than three years, three to five years, or more than five years, given that the USPSTF/ACOG/ASCCP guidelines consider a woman to be up-to-date on Pap testing within these intervals depending on age and the screening strategy that was utilized [22,23,24]. Participant’s Pap results were assessed with a multiple-choice question, “The last time you had a Pap test, did you get an “abnormal” result? In other words, did they say that they needed you to follow up for more testing?” and an open-ended question, “What were the results of your last Pap test?” Those participants who self-reported abnormal results were then asked about follow-up care. Women were given a multiple-choice question, “Did you get the recommended follow-up after your Pap test?” and then stratified according to whether they responded “yes” or “no.” Women with follow-up were asked additional questions about the type of follow-up care they received, and participants without follow-up were asked about reasons why they were unable to obtain recommended care [21,26,27,28,29,30].

To identify predictors for abnormal Pap follow-up, the survey assessed additional demographic information and variables that were found to have significance in previous studies of abnormal Pap follow-up in women with and without criminal-legal histories, including demographic information, duration of incarceration, access to a primary care provider, and provider communication [9,10,16,17,18,19]. All items were multiple choice or open-ended except for a question about efficacy of provider communication, which was scaled. In that question, participants were asked “My provider effectively explains information” according to the following four-item scale: all of the time, most of the time, some of the time, or never; for this item, a lower score indicates that providers were more likely to effectively explain information. Lesion severity, which has been associated with abnormal Pap follow-up in other studies, could not be assessed as we did not have access to medical records nor did the participants self-report their impression of whether or not their abnormal Pap tests were low or high grade [9]. All questions were adapted from validated surveys and designed to consider the unique needs of women with criminal-legal histories [21,26,27,28,29,30]. 

For data analysis, women in the cohort who reported abnormal results after their most recent Pap test were identified. Data on cervical cancer prevention behaviors, including Pap testing and follow-up, were stratified according to study site and compared using Chi-square for categorical variables and *t*-test for continuous variables. A *p*-value <0.05 was considered significant for bivariate comparisons. Descriptive statistics were used to describe types of follow-up care that women utilized or reasons why women did not obtain follow-up. Participants were then grouped according to whether they did or did not obtain abnormal Pap follow-up care. Independent variables included for comparison were demographic characteristics, social advantages (age, years incarcerated, education, employment, housing, health insurance, and healthcare provider), and perceptions of provider communication. We compared study groups with and without follow-up care using Chi-square for categorical variables and *t*-test for continuous variables with a *p*-value < 0.05 indicating statistical significance. After controlling for age, a multivariate analysis was performed to identify factors independently associated with obtaining follow-up care. Odds ratios (ORs) with Wald 95% confidence intervals (CIs) and corresponding *p*-values were reported. Statistical analysis was performed using SAS software, version 9.3 (SAS Institute Inc., Cary, NC, USA). 

## 3. Results

### 3.1. Description of the Entire Cohort 

There were *n* = 510 criminal-legal involved women in the cohort, which included *n* = 164 Birmingham, *n* = 108 Kansas City, and *n* = 238 Oakland women. The key demographic differences among the women in the three sites were age, race, and insurance status. The Kansas City women were younger (42.4 ± 11.7) compared to the other sites (Birmingham, 40.6 ± 11.3; Oakland, 45.4 ± 12.2; *p* < 0.001). The Oakland cohort was comprised of mostly black women (black, 80.7%; white, 4.6%), whereas the largest portions of women in Birmingham (black, 38.4%; white, 53.0%) and Kansas City (black, 34.3%; white, 43.5%) were white (*p* < 0.001). More criminal-legal women in Oakland had health insurance (94.5%) compared to women in Birmingham (39.0%) and Kansas City (51.9%, *p* < 0.001). There were no differences in educational attainment, employment/sources of money, sexual orientation, housing stability, living situation, cumulative time spent in jail/prison, history of violence, diagnosis of mental illness, or substance use across the study sites. In the cohort, there were *n* = 416 (82%) women with normal Pap results and *n* = 36 (7%) women who did not recall their Pap result. Additional demographic information by study site and data on women with normal Pap testing will be reported elsewhere. 

### 3.2. Description of Women with Abnormal Pap Testing

Within the cohort, there were *n* = 58 (11.4%) criminal-legal involved women who reported that their last Pap test was abnormal. The mean age of these women was 41.2 ± 9.9, and they consisted of 38% (*n* = 22) black, 52% (*n* = 30) white, 7% (*n* = 4) Hispanic, and 4% (*n* = 2) other women. More of the women with abnormal Pap results were from Kansas City (31%) and Birmingham (41%) than Oakland (28%, *p* = 0.003). Otherwise, there were no significant differences across the study sites, including receipt of abnormal Pap follow-up care. 

Among the women with abnormal Pap testing, the majority (62%) reported that their last Pap test was within 3 years. However, 24% reported a Pap test more than 5 years ago, suggesting that a large proportion of this group was not up-to-date on preventive cervical care. Most women had their Pap test as part of a routine well-woman exam (48%) and 12% reported a history of a prior abnormal Pap test results. Most women relied on a community clinic or health department (40%) for cervical cancer screening, but 17% relied on jail/prison-based screening programs (Table 1). 

Overall, 69% (*n* = 40) of criminal-legal involved women with abnormal Pap results obtained follow-up. Of those women, the majority received either repeat Pap/HPV co-testing (*n* = 15, 38%), or a colposcopy and/or biopsy (*n* = 14, 35%). Only *n* = 3 (8%) received a LEEP/ablative procedure or hysterectomy, respectively. There were *n* = 15 (26%) women who did not complete the follow-up, and the most commonly cited reasons were that they forgot (*n* = 5, 33%), lacked health insurance (*n* = 3, 20%), or were reincarcerated (*n* = 3, 20%; Table 2). The types of follow-up care and reasons for no follow-up care were compared across the study sites, but there were no significant differences (data not shown). There were also *n* = 3 (5%) with abnormal Pap results who did not provide any further details on their follow-up, and *n* = 3 (20%) whose appointment had not yet occurred. 

### 3.3. Predictors for Abnormal Pap Follow-Up Care

To identify predictors of follow-up care among criminal-legal involved women with abnormal Pap results, we compared the women who did and did not obtain follow-up (Table 3). Social advantages associated with abnormal Pap follow-up included older age (follow-up, 42.8 ± 10.6 years; no follow-up, 37.7 ± 7.1 years; *p* = 0.04) and having a primary care provider (follow-up, 67%; no follow-up, 33%; *p* = 0.005). Women who did not obtain follow-up were more likely to report full-time employment (15%, follow-up; 47%, no follow-up; *p* = 0.049). There were no significant associations between abnormal Pap follow-up and lifetime years incarcerated, education, housing, or health insurance status. 

Provider communication may also influence follow-up after an abnormal Pap result. Women who obtained follow-up were more likely to recall that they received information about the next steps in care (follow-up, 58%; no follow-up, 33%; *p* = 0.04). However, when comparing women with/without follow-up, according to the specific care plan they received (Pap testing or colposcopy/biopsy), there were no significant differences. Receiving specific information about HPV status or dysplasia also did not impact follow-up status. There was no difference in follow-up among those women who did not know or remember what they were told by their provider (follow-up, 8%; no follow-up, 20%; *p* = 0.36). In a multivariate analysis, both having a primary care provider (OR 4.6, 95% CI 1.3–16.0) and receiving specific communication about follow-up care (OR 3.8, 95% CI 1.1–13.2) were independently associated with abnormal Pap follow-up (Table 4). 

## 4. Discussion

Criminal-legal involved women are less likely to obtain preventive cervical care, and the current study provides insight into patient characteristics and provider practices that may facilitate follow-up among community-based women with abnormal Pap results. Overall, 69% of the women in the cohort obtained follow-up after an abnormal Pap result, which is similar to other reports of criminal-legal involved women, but much lower than the 90% follow-up rate observed in the general U.S. population [7,8,9,10]. Those criminal-legal involved women with older age and a primary care provider were more likely to obtain follow-up. We also found that specific communication from providers about next steps in care facilitated follow-up. Leveraging these variables—age, access to a provider, and provider communication—may offer an opportunity to mitigate the disparate incidence of cervical cancer among women with a history of criminal-legal involvement. Although, it is important to consider that criminal-legal involved women have complex lives and experience innumerable barriers to healthcare. Ensuring follow-up is likely dependent on each individual woman’s circumstances and needs. 

Having a community-based primary care provider was an independent predictor for abnormal Pap follow-up among criminal-legal involved women. Most jails and prisons do not coordinate health care once women are released, so providers and health systems must facilitate entry into community health care [31]. Many states with Medicaid expansion programs have developed strategies to enhance health care delivery to formerly incarcerated persons. These strategies include *data exchange,* so that a plan or provider is notified when someone is leaving jail or prison and re-entering the community; *jail or prison “in-reach*”, where a clinician meets with an inmate in person or via video conferencing to establish a relationship and develop a post-release care plan; *use of a peer support specialist,* who helps former inmates navigate health care and social service resources; and providing *specialized training to primary care providers* to help them better serve the unique needs of individuals with criminal-legal involvement [32]. These programs are certainly promising, but they are also state-dependent and only available in the 39 states that have adopted Medicaid expansion [32,33]. Furthermore, such programs may not necessarily emphasize cervical care. There is a need for more consistent linkages between criminal-legal involved women and community providers nationwide, as well as a need to promote cervical health in care delivery programs. 

Another key finding from the current study was the importance of specific provider communication regarding the follow-up plan. Those women who recalled information regarding the next steps in care were more likely to obtain follow-up, irrespective of what providers told them about their actual results or whether they needed a repeat Pap versus a colposcopy. Pap tests can take several weeks to result after they are collected, so it is important that providers have a plan for how they will communicate results and the next steps in care with criminal-legal involved women. Women in this population often have unstable housing, limited access to landlines, and frequent changes to their cell phone numbers [14,15]. Therefore, it would be prudent for providers to obtain multiple points of contact from patients, including contact information for friends or family members who may be able to reach them. The authors have also found success in maintaining contact with criminal-legal involved women through social media [34], which could be a consideration for those clinics that maintain professional social media accounts. Most of the women, both with and without abnormal Pap follow-up, reported that providers effectively explained information in their counseling. However, providers should be aware that criminal-legal involved women have lower health literacy, so it also important to make sure information is communicated using language that is clear and comprehensible [13,35,36].

There are no data on the relationship between age and cervical cancer prevention behaviors in criminal-legal involved women. In the general population, studies are conflicting. Some investigators have found no relationship with age, while others have found that either older or younger women are more likely to be compliant with preventive cervical care [17,18,19,37,38]. In a study by Menees et al, authors noted that older women were more likely to obtain recommended cancer screening, partially because providers were more likely to encourage cancer screening and follow-up care in older patients, and partially because the patients themselves perceived that they were at greater risk for a cancer diagnosis [37]. These assumptions are problematic because cervical cancer is most often diagnosed in younger women, aged 35–44 [39]. In our study, the mean age of women without follow-up was 37, which falls within the age range when cervical cancer is typically diagnosed. This finding highlights an opportunity to educate both providers and women with criminal-legal involvement about the age-associated risk for cervical cancer. 

Surprisingly, insurance status was not associated with abnormal Pap follow-up in the current study. Given the expansion of cervical cancer screening and diagnostic services for low-income and uninsured U.S. women, such as the CDC national breast and cervical cancer detection program, it is possible that women who seek follow-up are not necessarily relying on insurance to obtain care [40,41]. As states with Medicaid expansion are gaining expertise in enrolling criminal-legal involved persons into insurance plans, it has also become evident that insurance coverage does not necessarily equate to health care access [32,33]. It is equally as important to determine how to effectively deliver health care to criminal-legal involved women, considering the high rates of substance use, mental illness, and numerous social barriers that affect health behaviors in this population [5,13,42]. Because having a primary care provider and provider communication may have more influence on abnormal Pap follow-up than insurance status, interventions at the provider-level are critical to increasing health equity for criminal-legal involved women.

We expected full-time work to be a social advantage that would facilitate adherence to abnormal Pap follow-up, but our results suggest that work may be a barrier for criminal-legal involved women. Those criminal-legal involved women with full-time work have more reliable income and/or health insurance, but regular work may also limit their available time to seek preventive cervical care. Data from the U.S. national health interview study found that full-time workers were less likely to obtain preventive health services, including cervical cancer screening [43,44]. Health practitioners need innovative strategies to improve Pap follow-up adherence among criminal-legal involved women with full-time work. These women are often struggling to maintain housing, childcare, and other priorities, and may not have time available to seek cervical care during a typical clinic day [5,15]. 

Recidivism is another major barrier to preventive health care in the criminal-legal population [31]. In our study, most women who did not complete follow-up in our cohort cited that they either forgot or had not yet completed their appointment, but there were also several women who cited being reincarcerated. Given that over a half of women who are released from jail or prison are reincarcerated within three years, it is also important to expand access to cervical care within carceral settings [45]. In a study by Clarke et al., having on-site colposcopy significantly decreased the time to completing colposcopy among women with abnormal Pap testing [9]. While not all jails and prisons can offer more specialized women’s health services, such as colposcopy, these institutions should ensure that women with abnormal Pap testing are scheduled for follow-up care in another clinic or facility that can provide adequate, guideline-concordant cervical care. 

The major strength of this study is that it provides data from a multi-site cohort of women with criminal-legal histories. Because the participants live in cities with different health resource environments, the results may be more generalizable to criminal-legal involved women in other U.S. urban settings. An additional strength is that our data were drawn from women who are living in their community rather than jail or prison, which is rare in public health research, due to the challenges of maintaining consistent follow-up with women with criminal-legal involvement [46]. The primary limitation is that the data were cross sectional, so we do not know whether those women without follow-up ultimately received care; there were also *n* = 3 women whose appointment had not yet occurred, who may have also ultimately completed follow-up. A longitudinal data set would provide a more comprehensive analysis of health behaviors among criminal-legal involved women with abnormal Pap testing. Currently, the authors are collecting longitudinal data on the cohort and will publish the results as they become available. Another limitation is that we did not collect data from medical records to confirm abnormal results or assess the severity of dysplasia. Previous studies have found a high degree of correlation between self-reported Pap data and validated test results among criminal-legal involved women [47]. Finally, the 288-item survey takes approximately 45 minutes to complete and can be burdensome for participants. 

## 5. Conclusions

In conclusion, these data suggest that connecting criminal-legal involved women with providers in their community and ensuring providers offer tailored communication after an abnormal Pap test may help women obtain follow-up. Public health programs that link criminal-legal involved women with community health care, educate providers about the unique needs of this population, and accommodate women with full-time work may also facilitate abnormal Pap follow-up. Both providers and patients need education about the age-associated risk of cervical cancer to ensure that younger women with criminal-legal histories are receiving appropriate care. Recidivism may also be a barrier to follow-up, so criminal-legal involved women should receive appropriate social supports in outpatient settings that will reduce their risk of reincarceration and improve their connection to consistent healthcare. Criminal-legal involved women are at high risk for cervical dysplasia and cancer, so it is critical to give these women the assistance they need to facilitate life-saving preventive cervical care. 

## Figures and Tables

**Table 1:** Criminal-legal system involved women who reported abnormal results at time of last Pap test according to study site.

Variable	Total *n* = 58 (%)	Kansas City *n* = 18	Birmingham *n* = 24	Oakland *n* = 16	*p*-Value
Abnormal Pap (*n* = 58)	58 (100%)	18 (31.0)	24 (41.4)	16 (27.6)	0.0032
Timing of last Pap test					0.9032
<3 years ago	36 (62.0)	9 (50.0)	16 (66.7)	11 (68.8)	
3–5 years ago	5 (8.6)	1 (5.6)	2 (8.3)	2 (12.5)	
>5 years ago	14 (24.1)	5 (27.8)	6 (25.0)	3 (18.8)	
Unknown/prefer not to answer	3 (5.2)	3 (16.7)	0 (0)	0 (0)	
Reason for Pap test					0.1283
Well-woman or routine care	28 (48.3)	9 (50.0)	9 (37.5)	10 (62.5)	
Having problem/symptom	13 (22.4)	7 (38.9)	5 (20.8)	1 (6.3)	
Last Pap abnormal	7 (12.1)	1 (5.6)	3 (12.5)	3 (18.8)	
Screening at jail/prison	10 (17.2)	1 (5.6)	7 (29.2)	2 (12.5)	
Pap location					0.0559
Doctor’s office/clinic	18 (31.0)	9 (50.0)	8 (33.3)	1 (6.3)	
Community clinic/health dept	23 (39.7)	5 (27.8)	9 (37.5)	9 (56.3)	
Jail/prison	10 (17.2)	1 (5.6)	5 (20.8)	4 (25.0)	
Unknown/prefer not to answer	7 (12.0)	3 (16.7)	2 (8.3)	2 (12.5)	
Received follow-up	40 (69.0)	12 (66.7)	14 (58.3)	14 (87.5)	0.1437

**Table 2:** Abnormal Pap follow-up among criminal-legal involved women.

Variable	Total
Types of care among women who completed follow-up ^1^ (*n* = 40)	
Repeat Pap or HPV co-test	15 (37.5)
Colposcopy and/or biopsy	14 (35.0)
LEEP or ablative procedure	3 (7.5)
Hysterectomy	3 (7.5)
Unknown/prefer not to answer	5 (12.5)
Reasons for no care among women who did not follow up ^1^ (*n* = 15)	
Forgot	5 (33.3)
No transportation	1 (6.7)
No health insurance	3 (20.0)
Could not afford it	2 (13.3)
Reincarceration	3 (20.0)
My drug/alcohol abuse got in the way	1 (6.7)
Has not yet occurred	3 (20.0)

^1^ Participants were able to select multiple responses to this question.

**Table 3:** Variables associated with abnormal Pap follow-up among criminal-legal involved women.

Variable		Follow-Up *n* = 40 (%)	No Follow-Up *n* = 15 (%)	*p*-Value
**Social advantages**				
Mean Age		42.8 ± 10.6	37.7 ± 7.1	0.0358
Lifetime years incarcerated, mean (SD)		3.0 ± 3.8	4.6 ± 5.9	0.2993
More than high school education		9 (22.5)	7 (46.6)	0.1511
Full-time work		6 (15.0)	7 (46.6)	0.0493
Stable housing		27 (67.5)	15 (100.0)	0.3961
Health insurance		21 (52.5)	6 (40.0)	0.1494
Primary care provider		27 (67.5)	5 (33.3)	0.0049
**Provider communication**				
Efficacy of provider communication ^1^		1.4 ± 1.1	1.8 ± 0.3	0.1830
Participant’s recollection of provider communication following abnormal Pap result ^2^				
I was told nothing		1 (2.5)	2 (13.3)	0.2248
I was given specific information about HPV, cell changes, or cancer		18 (45.0)	6 (40.0)	0.4039
I was given specific information about the follow-up plan		23 (57.5)	5 (33.3)	0.0361
I was told I need a repeat Pap		11 (27.5)	2 (13.3)	0.1661
I was told I need a colposcopy/biopsy		17 (42.5)	3 (20.0)	0.0555
I don’t know/remember		3 (7.5)	3 (20.0)	0.3619

^1^ Lower score indicates more effective provider communication, ^2^ participants were able to select multiple responses to this question.

**Table 4:** Independent predictors for abnormal Pap follow-up ^1^.

Independent Variables	Odds Ratio (95% CI)	*p*-Value
Full-time work	0.34 (0.09, 1.28)	0.1112
Primary care provider	4.57 (1.30, 16.01)	0.0177
Specific information about follow-up care	3.78 (1.08, 13.21)	0.0381

^1^ Controlled for age.

## Data Availability

Supporting data from the baseline dataset can be made available upon completion of the study (2024) through a direct request to the corresponding author.
